# Putative Nociceptive Responses in a Decapod Crustacean: The Shore Crab (*Carcinus maenas*)

**DOI:** 10.3390/biology13110851

**Published:** 2024-10-22

**Authors:** Eleftherios Kasiouras, Peter C. Hubbard, Albin Gräns, Lynne U. Sneddon

**Affiliations:** 1 Department of Biological and Environmental Sciences, University of Gothenburg, P.O. Box 463, 405 31 Gothenburg, Sweden; lynne.sneddon@bioenv.gu.se; 2 Centre of Marine Sciences (CCMAR), Universidade do Algarve, Campus de Gambelas, 8005-139 Faro, Portugal; phubbard@ualg.pt; 3 Department of Applied Animal Science and Welfare, Swedish University of Agricultural Sciences, P.O. Box 463, 405 31 Gothenburg, Sweden; albin.grans@slu.se

**Keywords:** acetic acid, animal welfare, Decapoda, electrophysiology, pain

## Abstract

**Simple Summary:**

Nociceptors detect damaging stimuli and send signals to the central nervous system (CNS) about potential injury, which can give rise to pain. Crustaceans, such as shore crabs, are widely used in science and aquaculture. Understanding whether they can experience pain is essential for improving their welfare. One key criterion for assessing pain is the presence of nociceptors. This study investigated the existence of nociceptors in shore crabs by examining the CNS response to two types of potentially noxious stimuli, i.e., mechanical and chemical or acetic acid. Using electrophysiological equipment, the crabs’ CNS activity was measured when different parts of its body, such as the soft tissues of the claws, antennae, and legs were stimulated. The results suggest that the crabs responded to both mechanical and chemical stimuli, indicating the existence of putative nociceptors in these areas. Interestingly, responses to physical stimuli were shorter and more intense than the chemical stimuli, which elicited a longer response. The antennae responded only to the chemical stimuli with no discernable response to touch. Although further research is needed to fully understand pain in crustaceans, this study provides important information on the perception of tissue damage in a crustacean.

**Abstract:**

Nociceptors are receptors that detect injurious stimuli and are necessary to convey such information from the periphery to the central nervous system. While nociception has been extensively studied in various taxa, there is relatively little electrophysiological evidence for the existence of nociceptors in decapod crustaceans. This study investigated putative nociceptive responses in the shore crabs, specifically their response to mechanical and noxious chemical stimuli. Extracellular multi-unit electrophysiological recordings were conducted from the anterior ganglion and the circumesophageal connective ganglia to assess nociceptive responses. Soft tissues at the joints of the chelae, antennae, and walking legs were stimulated using acetic acid (noxious stimulus) and von Frey hairs (mechanical stimulus), while nearby ganglion activity was recorded. The results indicate the existence of nociceptors in the tested areas, with mechanical stimuli eliciting shorter, more intense neural activity compared with acetic acid. Although acetic acid triggered responses in all areas, the antennae and antennules did not respond to mechanical stimuli. Though we acknowledge the challenges of conducting in vivo electrophysiological recordings, future research should focus on further characterizing nociceptor activity because the results suggest the presence of nociceptors.

## 1. Introduction

Recent scientific evidence suggests that decapod crustaceans such as lobsters, crabs, prawns, crayfish, and shrimps may be able to experience pain [1,2]. However, information on the presence of nociceptors, i.e., receptors that detect noxious stimuli, remains limited. While nociceptive or pain-related behaviours have been observed [3], electrophysiological evidence of nociceptors is needed to identify which types of injurious stimuli may be painful. The existence of nociceptors alone does not necessarily mean that decapods experience pain [4], as other criteria must also be met to confirm that animals experience pain [5]. Nociception is a fundamental sensory system for the detection of stimuli that could or do damage to tissues [6]. When nociceptors are triggered by a stimulus, the signal can lead to the activation of dedicated neural circuits, initiating a protective withdrawal reflex, and in the case of injury, the signal can be transmitted to the central nervous system [7,8] where the experience of pain can arise [9]. Nociceptive pathways also connect to brain regions important for motivation, driving animals to evade potentially harmful stimuli, learning to avoid them in the future and protect themselves from further damage [5,10]. The advantage of nociception is clear, as it helps animals avoid injury and increases their chances of survival [5]. Additionally, animals may also experience an associated aversive motivational state akin to several aspects of pain observed in humans. Understanding the function of this state can guide us to refine the definition of pain and demonstrate its likelihood in specific animal taxa [5].

The capacity of an organism to experience pain can be assessed by monitoring the effects of a noxious stimulus on its neurobiology, physiology, and behaviour [5]. Understanding nociception and defining pain across taxa can have significant implications for the welfare of these organisms [11]. Interestingly there is some evidence of withdrawal reflexes, protective behaviours, and limping occurring after a potentially painful experience, suggesting the existence of nociceptors [12]. For instance, when acetic acid was brushed on the mouthparts of shore crabs, *Carcinus maenas*, there was a significant increase in grooming or rubbing in the affected area [13]. Moreover, when acetic acid was applied to the antennae of glass prawns, distinct behavioural patterns were observed compared to the norm; this was not significantly different when mechanical stimuli were applied, but behavioural observations of nociceptive reflexes were noticed [3]. Additionally, a recent study aimed to assess the behavioural responses of shore crabs when acetic acid was injected into the limbs [14]. Therefore, the presence of nociceptors, along with other criteria for assessing pain, reinforces the argument that these animals can detect injury-causing stimuli, meeting the criteria for animal pain [5].

Nociceptors have been identified in several invertebrates, including *Drosophila*, *C. elegans*, *Aplysia*, *Hirudo,* and others (see review in Sneddon et al., 2014) [5]. However, few studies have attempted to characterize nociceptors in decapods. Two studies used ex vivo recordings on the antennae of Louisiana red swamp crayfish (*Procambarus clarkia*) by applying water and chemical stimuli; hot water affected the isolated antennae, suggesting, possibly, the presence of thermal nociceptors [15,16]. Research on nociception has predominantly focused on mammals and other vertebrates [11]. There are two major classes of nociceptors: Aδ and C fibres that respond to mechanical, thermal, and/or chemical noxious stimuli [17]. Crabs have a condensed central nervous system consisting of several ganglia, with a brain located at the top of the head behind the eyes and a circumesophangeal connector linking the brain and the thoracic ganglia. Peripheral nerve roots from the thoracic ganglia innervate the crab’s legs [18]. While researchers have hypothesized about the function of nociceptors in crustaceans, physiological data remain limited [11]. It has been speculated that nociceptors in crustaceans could be detectable by extracellular recordings, likely involving tonic excitatory neurons responsive to concentrated acidic or basic solutions [3].

The present study investigates the existence of nociceptive responses in various soft tissues across the bodies of shore crabs (*Carcinus maenas*). This species was chosen as a model decapod due to its widespread distribution [19] and well-documented physiology [20]. Electrophysiological recordings were conducted on the brain or the circumesophageal ganglion in response to mechanical and noxious stimuli. Soft tissue areas, including the eyes, the antennae, antennules, the soft tissue between the claws, and the soft tissues at the joints of the pereiopods, were stimulated using von Frey hairs or acetic acid. We hypothesize that, if nociceptors are present in the soft tissues of shore crabs, then the stimulation of these tissues with von Frey hairs or acetic acid will elicit detectable electrophysiological responses in the brain or the circumesophageal ganglion. These responses will be consistent with the activation of nociceptive pathways, suggesting that these tissues contain nociceptors capable of responding to mechanical and chemical noxious stimuli. Additionally, we hypothesize that if the electrophysiological recordings from the brain and circumesophageal ganglion of shore crabs reveal differential responses to mechanical versus chemical stimuli, then it can be inferred that nociceptors in these tissues may have distinct modalities for detecting various types of noxious stimuli. This would indicate that shore crabs possess specialized nociceptive mechanisms for processing different types of harmful stimuli.

## 2. Materials and Methods

### 2.1. Ethical Considerations

Although decapod crustaceans are not currently protected under the European Directive 2010/63/EU, this study aimed to minimize the number of animals used and ensure their welfare during the experiment. Thus, only a relatively small number of animals were used to provide evidence of their capacity for potentially painful stimuli to be conveyed to the CNS, which could support their inclusion in future animal welfare legislation, thereby improving the welfare of many decapod crustaceans. Animal welfare was monitored twice daily, and animals were housed in appropriate conditions as described below.

### 2.2. Animals

Shore crabs (*Carcinus maenas*) (mean: 13.95 ± 1.31 g, n = 20) were collected using creels from Ria Formosa, Portugal. They were initially housed at the Ramalhete Marine Station (Ria Formosa Natural Park, Faro, Portugal) in a 1000 L tank (110 × 110 × 80 cm) at 21 °C with natural seawater (35 ppt) supplied through an open circuit, which was sand-filtered and UV-treated. The tank was outside under a natural photoperiod of 13 h light and 11 h dark. Crabs were fed mussels (*Mytilus* spp.) three times per week and were acclimated to this environment for two weeks prior to experimentations. Around 20 crabs were then transported in a 25 L bucket with aerated seawater to the laboratory at the CCMAR Gambelas campus, a 20 min drive away. All crabs were in good health upon arrival. In the laboratory, crabs were transferred to a glass tank (60 × 40 × 60 cm) with an opaque cover containing aerated natural seawater (temperature = 18 ± 1 °C, pH = 8 ± 0.1, NH4 < 0.1 mg/L, NO_2_ < 0.1 mg/L, NO_3_ < 20 mg/L). The tank was equipped with stones, pebbles, and terracotta pots to provide hiding spaces and cover. The temperature was maintained at 18 ± 1 °C, and the room had an ambient light cycle. Crabs were held in these conditions from 2 to 7 days before the experiments. During this period, they were fed ad libitum with mussels twice a week, with feeding occurring at least 24 h before experiments. One-third of the seawater was replaced after every feed.

### 2.3. Experimental Protocol

Ringer’s solution was prepared to dilute the neuromuscular blockers and maintain the tissue viability during experiments. The solution comprised the following: 470 mM NaCl, 7.9 mM KCl, 15.0 mM CaCl_2_·2H_2_O, 6.98 mM MgCl_2_·6H_2_O, 11.0 mM dextrose, 5 mM HEPES acid, and 5 mM HEPES base adjusted to pH 7.5 [21]. The neuromuscular blockers were 4-Aminopyridine (4-AP) and tetraethylammonium (TEA). These neuromuscular blockers function as potassium blockers, but Tanner et al., in 2022, found that when both blockers are used in combination with low concentrations, there are no additional effects on sensory neurons [21]. Thus, 4-AP and TEA were added to 10 mL of Ringer’s solution at 50 mM and 200 mM, respectively. These concentrations effectively immobilized the crabs for two hours, rendering their limbs and claws immobile while allowing movement of the mouthparts. The use of neuromuscular blockers facilitated electrode implantation and uninterrupted recordings. Anesthetics were not used to avoid any potential dampening of CNS responses due to sedation.

**Figure 1:**
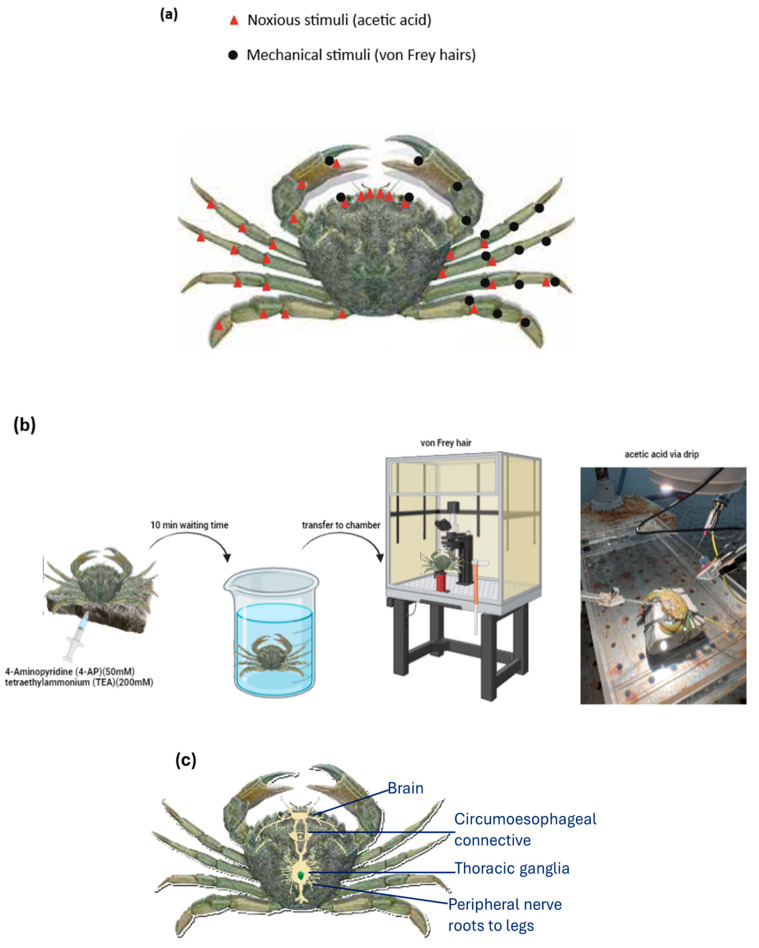
(**a**) Areas on the shore crabs (*Carcinus maenas*) where acetic acid and von Frey hair were applied to investigate the presence of nociceptors. The tissues examined included the eyes, the antennae, the antennules, the soft tissue between the claws, and the soft tissue at the joints of the pereiopods (n = 20). (**b**) A schematic of the timeline of the experimental protocol and areas where acetic acid or mechanical stimuli were applied on the shore crab. Initially, animals received an injection of neuromuscular blockers. Then, after the waiting time was over for the blockers to reach a full effect, animals were transferred into the chamber where electrophysiological recordings took place (copyright: picture of a shore crab, Victorian Fisheries Authority, https://vfa.vic.gov.au/operational-policy/pests-and-diseases/noxious-aquatic-species-in-victoria/european-shore-crab (accessed on 19 September 2024)). (**c**) A schematic of the nervous system of a shore crab (*Carcinus maenas*).

Crabs were randomly selected from their tank in the laboratory, and all 20 crabs were used by the end of the experiments. Shore crabs received an injection into the body via the soft tissue between the carapace and 5th leg with neuromuscular blockers (injection volume: 0.1 mL), which took approximately 10 min to reach their full effect where crabs were unable to move and did not react when approached with a net. Each crab was then secured with rubber bands onto a flat stone, ensuring that its body remained submerged in seawater to allow respiration while the upper carapace was exposed for access to various ganglionic areas. Recordings were made from different regions of the brain ganglion and the circumesophageal connective ganglion. To expose these areas, a drill was used to cut a small window into the carapace without damaging the underlying tissues. Stimuli applied included acetic acid at concentrations of 0.1%, 0.5%, 1%, and 5%, or von Frey hairs of 1.0 g, 0.16 g, 0.04 g, and 0.008 g. In addition, measurements were repeated in the same areas with the same stimulation to validate the responses (Tables S1–S3). Stimuli, such as 0.5 mM glutamate dissolved in seawater and mussel juice (mashed-up mussels in seawater), were also tested to distinguish between chemoreceptive and nociceptive responses; however, no responses were observed for these stimuli. Tungsten micro-electrodes (0.1 MΩ, World Precision Instruments, Freidberg, Germany, www.wpi-europe.com/index.aspx (accessed on 22 May 2023) were placed in the ganglion receiving input from the stimulated area. For eye stimulation, electrodes were positioned on the optic lobes of the brain ganglion according to the neuroanatomy schematics of Abbot (1971) and Krieger et al. (2012) [22,23]. For the antennae and antennules, electrodes were placed on the olfactory lobe of the brain ganglion following the same schematics. When stimulating the soft tissues of the claws or the soft tissue of the limb joints, the electrodes were placed on the circumesophageal connective. The stimulated areas are illustrated in Figure 1a. Not all areas were stimulated in the same animal due to difficulties in maintaining the animals for prolonged periods. Usually, recordings in one animal lasted approximately 2–3 h. Initially, the electrodes were correctly positioned in the relevant area, and then acetic acid and/or von Frey hair were tested, i.e., on the eyes. Usually, the first stimulus that was applied was either von Frey hair or a low concentration of acetic acid. Then, a control stimulus was applied, and then various concentrations of acetic acid or von Frey hair were tested in the specific areas (Figure 1b). This order was followed to avoid rendering the receptors unresponsive because when a high concentration of acetic acid was used before any other stimuli, no further stimulation in the area was possible, suggesting nerve damage.

The crabs were earthed with a copper wire inserted in the telson at the posterior end. The raw signal was amplified (×20,000; AC pre-amplifier, Neurolog NL104, Digitimer Ltd., Welwyn Garden City, UK; www.digitimer.com/ (accessed on 22 May 2024)), filtered (high pass: 200 Hz, low pass: 3000 Hz; Neurolog NL125, Digitimer Ltd.) and integrated (time constant 1 s; Neurolog NL703, Digitimer Ltd.) similarly to Velez et al., 2024 [24]. Raw and integrated signals were digitized (Digidata 1440A, Molecular Devices, San Jose, CA, USA, www.moleculardevices.com/ (accessed on 17 May 2023)) and recorded on a PC running AxoScope software (version 10.6, Molecular Devices). Data analyses, including peak count and amplitude measurements of the integrated responses, were performed using Axoscope Software. Moreover, for the distinction of true and non-true responses, a similar methodology was followed by Velez et al., 2024 [24].

### 2.4. Statistical Analysis

Data were analyzed in AxoScope software (version 10.6, Molecular Devices) to compare the responses between stimuli and assess which responses to acetic acid or mechanical stimulation were valid responses in comparison to a control (no stimuli) [24]. Furthermore, to assess any potential differences between the amplitudes from the integrated responses received by various concentrations of acetic acid and pressure applied by the von Frey hairs, the data were analyzed using SPSS version 29.0 (IBM Corp., Armonk, NY, USA). The comparison was between units of meaning and the number of responses received. The normality of the amplitudes from the tissue stimulation with acetic acid and von Frey hairs was assessed using a Shapiro–Wilk test. As the data were non-normally distributed, non-parametric tests were employed to compare the amplitudes between stimuli. Specifically, the different concentrations of acetic acid stimulations in the eyes and antennules were compared using a Kruskal–Wallis test followed by Dunn’s pairwise comparison. For comparisons of amplitudes between different concentrations of acetic acid in each region (the claws, the antennae, and the legs) were compared separately with a Mann–Whitney U-test. Also, the different amplitudes from the pressure levels of the von Frey hair stimulation of the legs and claws were analyzed using a Mann–Whitney U-test. Additionally, the compiled maximum amplitude and the duration of data for the mechanical and chemical stimuli were compared with a Mann–Whitney U-test. Data are presented as box plots exhibiting the median ranging from min to max (^ns^ *p* > 0.05; * *p* < 0.05; ** *p* < 0.01; *** *p* < 0.001). Finally, the stimuli were compared across different tissues within some individuals with a Wilcoxon test.

## 3. Results

**Figure 2:**
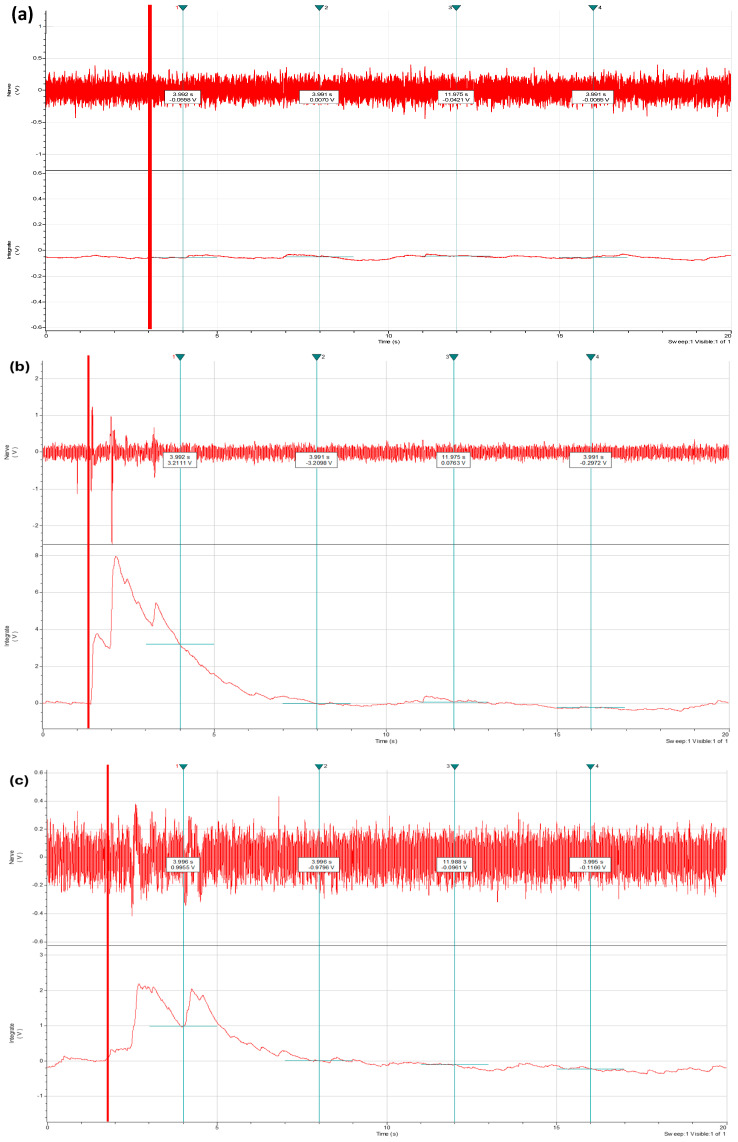
(**a**) Raw trace of response when seawater was applied on the legs; (**b**) raw trace of response when von Frey hair was applied on the claws; and (**c**) raw trace of response when acetic acid was applied on the legs (shore crabs, n = 20). The red vertical line represents the moment that the stimulus was introduced.

The number of responses recorded in the central nervous system per stimulated area is exhibited in Table 1. Activity in the brain ganglion demonstrated that stimulating the eye, antennae, and antennules with acetic acid elicited a response (Figure 2). Similarly, activity recorded in the circumeshophageal connective and the brain ganglion was observed during the stimulation of the intersegmental bundles in walking legs and claws.

### 3.1. Maximum Amplitude and Duration of Stimulus Between Mechanical and Acetic Acid

Areas that elicited a response reacted to both stimuli, but when compared with a Mann–Whitney U test, noxious acetic acid was characterized by a higher duration and lower amplitude response. In comparison, the mechanical stimulus yielded a response that was shorter with higher amplitude (duration of amplitude: Z = −8.793, *p*-value < 0.001, maximum amplitude: Z = −2.563, *p*-value = 0.010).

### 3.2. Eyes

**Figure 3:**
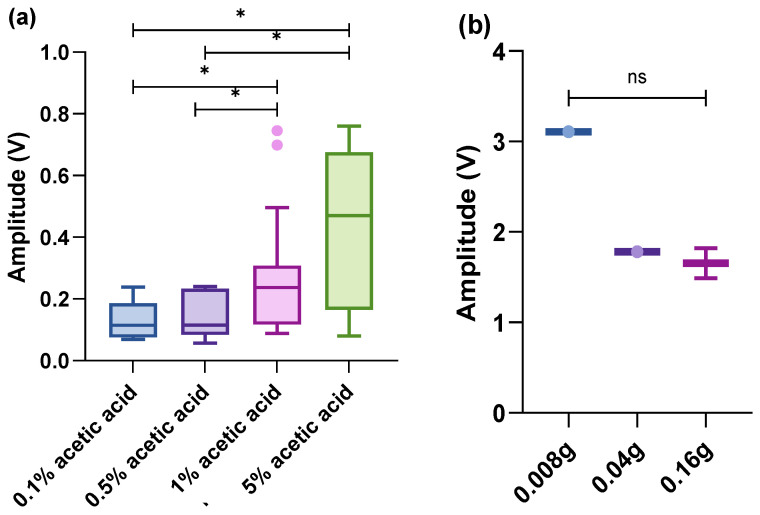
Box plot (within the box, the horizontal lines denote the median values; boxes extend from the 25th to 75th percentiles of each group’s values; the vertical extended lines represent the 95% range of values; and dots denote the outliers). A comparison of integrated amplitudes of the maximum amplitude per treatment. (**a**) Noxious stimulus (acetic acid) on the eyes (n = 35, responses); (**b**) mechanical stimulus (von Frey hairs) on the eyes (n = 4, responses) (^ns^ *p* > 0.05; * *p* < 0.05).

During acetic acid stimulation, concentration-dependent differences were observed. The amplitude was significantly higher when the eyes were stimulated with 5% acetic acid compared to 1%, 0.5% and 0.1% (H = 9.514, *p*-value = 0.023. Comparisons 5%–1% *p*-value = 0.42; 5%–0.5% *p*-value = 0.026; 5%–0.1% *p*-value = 0.019; 1%–0.5% *p*-value = 0.047; 1%–0.1% *p*-value = 0.034; 0.5%–0.1% *p*-value = 0.835) (Figure 3a). Mechanical stimuli elicited a response with a much higher amplitude than the acid; however, there appeared to be no differences between 0.008, 0.04, and 0.16 g (H = 1.800, *p*-value = 0.407), although the duration of the mechanical stimuli was shorter than the acetic acid (Figure 3b).

### 3.3. Limbs

**Figure 4:**
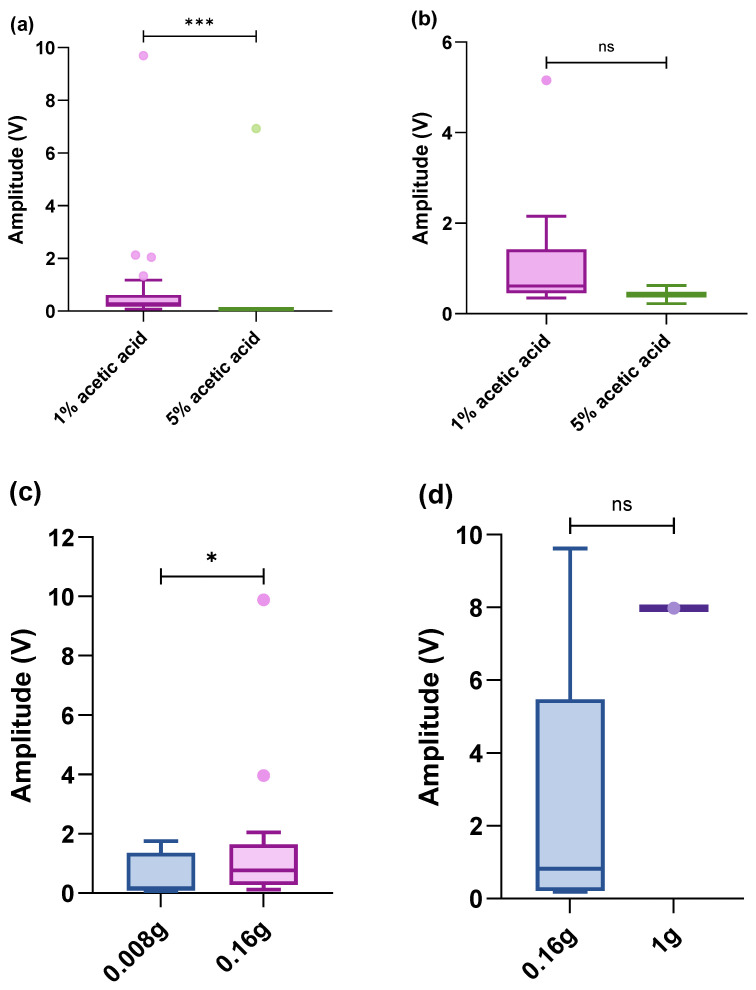
Box-plot (within the box, the horizontal lines denote the median values; boxes extend from the 25th to 75th percentiles of each group’s values; the vertical extended lines represent the 95% range of values; dots denote the outliers). Comparison of integrated amplitudes of the maximum amplitude per treatment after acetic acid on the (**a**) legs (n = 47, responses) and (**b**) claws (n = 15, responses). (**c**) Mechanical stimulus (von Frey hair) on the legs (n = 24, responses) and (**d**) on the claws (n = 6, responses) (^ns^ *p* > 0.05; * *p* < 0.05; *** *p* < 0.001).

When stimulating the inter-segmental membranes of walking legs and claws, 1% acetic acid evoked a significantly higher amplitude of response than 5% acetic acid (legs: Z = −3.284, *p*-value < 0.001; claws: Z = −1.019, *p*-value = 0.308). Additionally, the largest amplitude of acetic acid stimulation was lower and the duration of response was longer than mechanical stimulation (Figure 4a,b). Additionally, results from the mechanical stimuli in these areas showed that the pressure applied to the tissues increased with the response amplitude (legs: Z = −1.960, *p*-value = 0.05; claws: Z = −0.878, *p*-value = 0.380) (Figure 4c,d).

### 3.4. Antennae and Antennules

**Figure 5:**
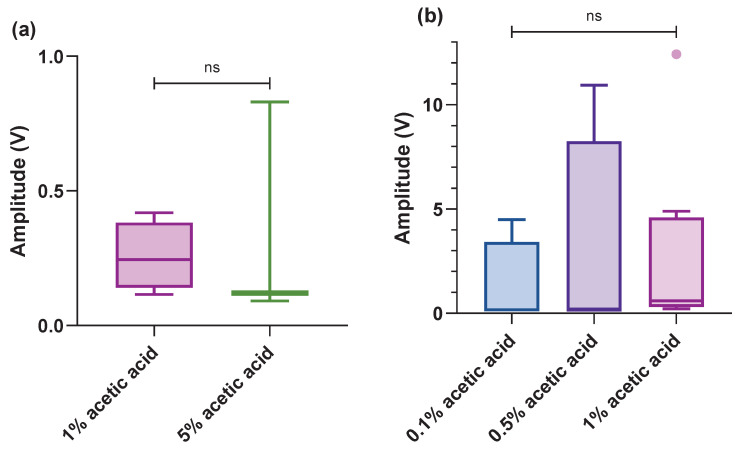
Box plot (within the box, the horizontal lines denote the median values; boxes extend from the 25th to 75th percentiles of each group’s values; the vertical extended lines represent the 95% range of values; dots denote the outliers). A comparison of integrated amplitudes of the maximum amplitude per treatment after acetic acid on the (**a**) antennae (n = 7, responses); (**b**) acetic acid on the antennules (n = 17, responses) (^ns^ *p* > 0.05).

Only two concentrations of acetic acid seemed to have a strong effect on ganglionic activity on the antennae, with no difference in amplitude between 1 and 5% (Z = −0.354, *p*-value = 0.724). Additionally, on the antennules, 1% appeared to evoke a significantly stronger response than 0.5% and 0.1% acetic acid, but this was not significantly different (H = 4.127, *p*-value = 0.127). However, there was no response to mechanical stimuli in these areas (Figure 5a,b).

### 3.5. Comparison of Responses Across Tissues Within Individuals

Firstly, the responses from two mechanical stimuli on the legs (0.16 g and 0.08 g) were compared within one individual, and there were no significant differences (Z = −1.604, *p*-value = 0.109). The rest of the comparisons were from responses to acetic acid stimuli. Two different stimuli were compared for the antennae (1% and 5%), and there were no differences (Z = −1.342, *p*-value = 0.180). Then, three different stimuli were compared on the eyes (0.1%, 0.5%, and 1% acetic acid), and there were also no differences (0.1%–0.5% Z = −0.447, *p*-value = 0.655; 0.1%–1% Z = −1.342, *p*-value = 0.180; 0.5%–1% Z = −1.342, *p*-value = 0.180). Within another individual, the same comparison on the eyes was conducted, and there were no significant differences (0.1%–0.5% Z = −0.447, *p*-value = 0.655; 0.1%–1% Z = −0.447, *p*-value = 0.655; 0.5%–1% Z = −0.535, *p*-value = 0.593). Finally, these three concentrations of acetic acid were compared on the antennules, but no statistically significant differences were noticed (0.1%–0.5% Z = −1.342, *p*-value = 0.180; 0.1%–1% Z = −1.342, *p*-value = 0.180; 0.5%–1% Z = −1.342, *p*-value = 0.180).

## 4. Discussion

To our knowledge, this study is the first to report in vivo electrophysiological recordings of putative nociceptive responses in crabs. Our results suggest the presence of nociceptive activity in shore crabs as they respond to acetic acid, a standard pain test in vertebrates including fishes (e.g., Sneddon et al., 2004 [25] amphibians, reptiles, birds, and mammals [26,27]). Nociceptors are defined as sensory receptors that preferentially respond to potentially harmful stimuli [28,29]. Our findings in this study show the possibility that shore crabs are capable of nociception, as the results exhibited how shore crabs respond to both mechanical stimulation and noxious chemicals, such as acetic acid, with varying intensities. These stimuli were conveyed to the central nervous system from a variety of soft tissues around the crab’s body, showing that shore crabs can detect both mechanical stimuli and noxious acetic acid [30].

The mechanical thresholds for stimulating these tissues were notably lower than those found in other taxa. For example, tissues such as the eyes and the soft tissues of the walking legs had a threshold as low as 0.008 g, while the claws had a threshold of 0.16 g. The leg threshold is considerably lower than those observed in the nociceptors of fish (0.1 g) [25] and much lower than in humans (0.6 g) [31]. Claws are used to crush molluscan shells during feeding [32], so, in terms of function, it would seem intuitive that the claws had higher mechanical thresholds compared with walking legs. The compound eyes of shore crabs, composed of hundreds of thousands of light sensors with their own lenses and corneas [33], are particularly delicate, which may account for the lower mechanical stimulation threshold due to their susceptibility to damage. Similarly, the soft tissues of the pereopods exhibited the same threshold as the eyes, suggesting that these tissues between the joints are sensitive to external mechanical stimuli. In contrast, the joints of the claws responded only to higher mechanical stimuli, comparable to the levels observed when stimulating nociceptors on the skin of rainbow trout [25]. Additionally, von Frey hairs were applied to the antennae and antennules, but no responses were detected. Previous research has shown that antennules can sense various stimuli, including mechanical and both noxious and non-noxious chemical stimuli [15,34]. The lack of response in our study might be due to these areas having higher mechanical thresholds than those used in the present study. Alternatively, they may be mechanically insensitive and perform a largely chemosensory function. However, a behavioural study in glass prawns revealed that while these animals responded to noxious stimuli, the mechanical stimulation of their antennae did not trigger grooming [3]. This suggests that the antennae have a high threshold for mechanical stimuli, which agrees with the findings of this study. Furthermore, in some animals, acetic acid was introduced before mechanical stimuli, potentially damaging the receptors and rendering them unresponsive to further stimuli [35]. However, some animals did not respond when mechanical stimulation was applied first, which might mean that antennae and antennules do not possess mechanoreceptors. Chemonociceptors have been identified in mammals, and thus, it is possible that the receptors in these areas just simply do not have mechanical sensitivity [36]. More detailed studies are needed to address the functionality of receptors on antennae and antennules.

When the different tissues of shore crabs were stimulated with acetic acid, the eyes and antennules had the lowest thresholds, which responded to a concentration of 0.1% acetic acid. In contrast, the walking legs, claws, and antennae had a higher threshold, responding to 1% acetic acid. The increased sensitivity of the eyes and antennules can be attributed to both their comparatively delicate structure and, in the case of the antennules, their chemosensory function, although this is mainly linked to non-noxious chemoreception [34]. Previous studies in frogs have demonstrated differences in nociceptive responses to varying concentrations of acetic acid, which agree with our findings [37]. The higher the acid concentration, the greater the amplitude of the response. However, when the tissues were stimulated with 5% acetic acid, the amplitude of the stimuli was lower compared to when 1% acetic acid was used; this could be attributed to the stimulus being so strong that damage occurred to the receptors, subsequently reducing the input to the CNS. This is supported by attempts to repeat the stimulation in the same area after 5% acetic acid failed to evoke any response. Moreover, behavioural observations demonstrated that when shore crabs were exposed to acetic acid on their mouth and eyes, there was increased movement of the mouthparts and an attempt to hold down the eye treated with acetic acid [13]. Other behavioural studies have documented protective and escapist behaviours in response to potentially painful stimuli [12]. Concerns have been raised over the replicability of the responses to acetic acid stimulation in decapod crustaceans, but the results of the present study and those of other studies demonstrate consistent responses across different laboratories and species [3,13,14,38]. These behavioural observations, combined with the electrophysiological evidence from this study, strengthen the argument for the existence of nociception in decapod crustaceans, which is a key piece of evidence for the possibility of pain.

The shore crab’s central nervous system (CNS) activity showed a higher amplitude of response to mechanical stimulation compared to acetic acid. However, the duration of CNS activity was longer when these areas were exposed to acetic acid. This demonstrates the specificity of CNS’s response where the mechanical and chemical employed here have a different coding pattern and, as such, can be differentiated from one another, which has been observed in other animals [28,39]. Vertebrate polymodal nociceptors typically show the same amplitude to different noxious stimuli but may alter the firing rate depending on whether they are responding to thermal, chemical, or noxious stimuli [40]. These nociceptors also show a slowly adapting response to mechanical stimulation, firing for the duration of the stimulus, whereas touch and pressure receptors exhibit a fast-adapting response where the receptor fires rapidly and stops quickly [17,40]. Thus, it is likely that the receptors for mechanical stimulation differ from those responding to acetic acid, which agrees with a previous study conducted on glass prawns [3]. It is not possible to know for certain from the present and previous studies whether the responses to mechanical stimuli were touch responses or noxious. Thus, further studies are needed to clearly define whether this type of mechanical stimulation is truly nociceptor mediated. The longer duration of response to acid may be linked to the nature of the noxious stimulus itself since the application of acid is likely to excite the free nerve endings of any nociceptors present for a longer duration than the more rapid application of a von Frey hair to the same area.

In all tissues tested, there was a concentration-dependent response with acetic acid, typically increasing up to 1% acetic acid, which elicited a relatively stronger response in the CNS. Chemosensory receptors and nociceptors in other animals classically responded in this manner [5,36]. When 5% acetic acid was applied, it appeared to damage the area, thereby silencing the response. Moreover, receptors stimulated with 5% acetic acid failed to respond to subsequent stimuli, indicating potential tissue damage and rendering the receptors non-responsive [41]. Similar thresholds for 1% acetic acid have also been observed in rainbow trout [42], with 2% or higher resulting in tissue damage where nociceptors gave a burst of activity and then fell silent. Only the eyes and antennules responded to lower concentrations of acetic acid (0.1% and 0.5%), while receptors in other areas did not. These lower thresholds may be due to the delicate structures of these tissues, but antennules also have at least four sets of setae, which are sensitive to chemical stimuli [15,43]. In response to mechanical stimulation, the eyes exhibited sensitivity to a pressure of 0.008 g of hairs, a threshold lower than that found in trout (0.1 g) [25] or in humans (0.6 g) [31]. Interestingly, receptors on the leg joints also responded to 0.008 g, while those on the claws had a higher threshold of 0.16 g. Thus, the function of these body areas may affect the thresholds of noxious and mechanical stimuli, as seen in other species. Both antennae and antennules contribute to chemosensory, and it has been shown that high temperatures elicit an intense response on the antennae. Additionally, the antennules, due to their structure, are extremely sensitive to chemical and mechanical stimulations [15,30,43].

Ultimately, given the novelty of this study, several considerations and caveats are necessary. Ideally, future studies should use single-unit extracellular recordings to characterize individual receptive fields and their properties. However, we found that the majority of electrophysiological studies are conducted ex vivo with neuronal tissues removed into Ringer’s solution, which is valuable but does not allow in vivo responses to be recorded. Improved experimental protocols that ensure the animals remain viable for extended periods are necessary because, in this study, animals started to deteriorate after two hours and so were humanely killed. This does prevent longer-term monitoring of the nervous system in the intact animal. Moreover, new filters and different frequencies of the electrophysiological recording equipment should be adjusted to better describe the characteristics of nociceptors, including the size of the receptive fields. Single unit recordings to determine the accurate properties of the action potentials and developing methods to calculate the speed of transduction or conduction velocity will also help clearly define nociceptors in decapods. Finally, future studies should build upon this work to fully define nociceptors by testing other noxious stimuli, such as extremes of cold and heat, alongside modulation using analgesic chemicals.

## 5. Conclusions

These results indicate that 32 areas around the crab’s body exhibited putative nociceptive responses that respond to a noxious chemical, with many areas having additional mechanical sensitivity. Further research is needed to clearly define the properties of nociceptors in this species and other decapod crustaceans. However, this is an important first step in identifying whether nociceptors exist in shore crabs and how information is conveyed to the CNS. Notably, the present study is one of the first empirical electrophysiological studies that demonstrate the existence of putative nociceptive responses in various body areas of a live decapod crustacean. The capacity for pain in these animals has been questioned, and this study provides further evidence that can be used to determine the welfare implications and humane treatment of decapod crustaceans.

## Figures and Tables

**Table 1:** Recorded activity in the brain ganglion or circumesophageal connective ganglion of shore crabs (*Carcinus maenas*) following the stimulation of various soft tissue areas with either mechanical stimuli or acetic acid (n = 20).

Responses (Number)		
Areas	Mechanical	Acetic
Eyes	4	35
Legs	24	47
Claws	6	15
Antennae	0	7
Antennules	0	17
Total	34	121

## Data Availability

The raw data and Supplementary Tables are available at figshare.com (accessed on 13 October 2024), https://figshare.com/s/f28e5608a4fb6412506e; 10.6084/m9.figshare.27052978.

## Supplementary Materials

The following supporting information can be downloaded at: https://www.mdpi.com/article/10.3390/biology13110851/s1, Table S1: Recorded activity in the brain ganglion of shore crabs (*Carcinus maenas*) following stimulation of various soft tissue areas with acetic acid (n = 20). Summary of responses (units) received from each animal; Table S2: Recorded activity in the brain ganglion of shore crabs (*Carcinus maenas*) following stimulation of various soft tissue areas with von Frey hair (n = 20). Summary of responses (units) received from each animal; Table S3: Recorded activity in the brain ganglion of shore crabs (*Carcinus maenas*) following stimulation of various soft tissue areas with von Frey hair (n = 20). Summary of responses (units) received from each animal. Eyes: glutamic acid and the light were considered as positive control; negative control was the sea water. Antennules: glutamic acid, mussel juice were considered olfactory stimuli; negative control the sea water and hot sea water as thermal stimuli. Legs: mussels’ juice was tested as olfactory stimuli on dactylopodite (5^TH^ leg last segment) and seawater as negative control. Carapace: mechanical stimuli were tested on carapace as negative control.
