# Supplementary material for: Putative Nociceptive Responses in a Decapod Crustacean: The Shore Crab (Carcinus maenas)
Source: Biology (Basel). 2024 Oct 22;13(11):851. doi: 10.3390/biology13110851 (PMC11591628; doi:10.3390/biology13110851)
Supplement: Supplementary file 1 [file biology-13-00851-s001.zip › biology-3242451-supplementary.pdf]

Supplementary:

Table S1. Recorded activity in the brain ganglion of shore crabs (*Carcinus maenas*) following stimulation of various soft tissue areas with acetic acid (n=20). Summary of responses (units) received from each animal.

Number of stimuli

Acetic acid (noxious)

Animal no.	Eyes				Legs				Claws				Antennas				Antennules			
	0.1 %	0.5 %	1%	5 %	0.1 %	0.5 %	1%	5 %	0.1 %	0.5 %	1%	5 %	0.1 %	0.5 %	1 %	5 %	0.1 %	0.5 %	1 %	5 %
1							3													
2																				
3			1	5								2			3	2				
4								8								1			2	
5			3																	
6	2	2	2																	
7	2	3	5																	
8	1		1				6													
9							2													
10			4				2													
11																	1	1	2	
12							3													
13							5				4									
14							6				7									
15																	1	1	1	
16	1	2	1												1		2	2	1	
17																			3	
18							2				2									
19																				
20							10													
Total no.	6	7	17	5	-	-	39	8	-	-	13	2	-	-	4	3	4	4	9	-

Table S2. Recorded activity in the brain ganglion of shore crabs (*Carcinus maenas*) following stimulation of various soft tissue areas with von Frey hair (n=20). Summary of responses (units) received from each animal.

Number of stimuli

von Frey hair (mechanical)

Animal no.	Eyes				Legs			Claws			Antennas			Antennules		
	0.008 g	0.16 g	0.0 4g	1 g	0.008 g	0.16 g	1 g	0.008 g	0.16 g	1 g	0.008 g	0.16 g	1 g	0.008 g	0.16 g	1 g
1						4			2							
2					6	3			1							
3																

4																
5																
6																
7																
8																
9					1	5			2	1						
10	1	2	1		1	4										
11																
12																
13																
14																
15																
16																
17																
18																
19																
20																
Total no.	1	2	1	-	8	16	-	-	5	1	-	-	-	-	-	-

Table S3. Recorded activity in the brain ganglion of shore crabs (*Carcinus maenas*) following stimulation of various soft tissue areas with von Frey hair (n=20). Summary of responses (units) received from each animal. Eyes: glutamic acid and the light were considered as positive control; negative control was the sea water. Antennules: glutamic acid, mussel juice were considered olfactory stimuli; negative control the sea water and hot sea water as thermal stimuli. Legs: mussels' juice was tested as olfactory stimuli on dactylopodite (5TH leg last segment) and seawater as negative control. Carapace: mechanical stimuli were tested on carapace as negative control.

	Number of stimuli									
	Positive & negative control/ Olfactory/ Thermal									Mechanical
Animal no.	Eyes			Antennules				Legs		Carapace
	10^{-3} M glutamic acid	sea water	light	10^{-3} M glutamic acid	mussel juice	sea water	hot sea water	musel juice	sea water	
1										
2										
3	1	2	1							
4				2						
5				1						
6		2								
7										
8										
9										2
10									1	
11										

12										
13										
14										
15										
16										
17										
18										
19					2	2	3			
20								1		
Total no.	1	4	1	3	2	2	3	1	1	2
